# Supplementary figures and images for: Validity of the models predicting 10-year risk of cardiovascular diseases in Asia: A systematic review and prediction model meta-analysis
Source: PLoS One. 2023 Nov 30;18(11):e0292396. doi: 10.1371/journal.pone.0292396 (PMC10688732; doi:10.1371/journal.pone.0292396)

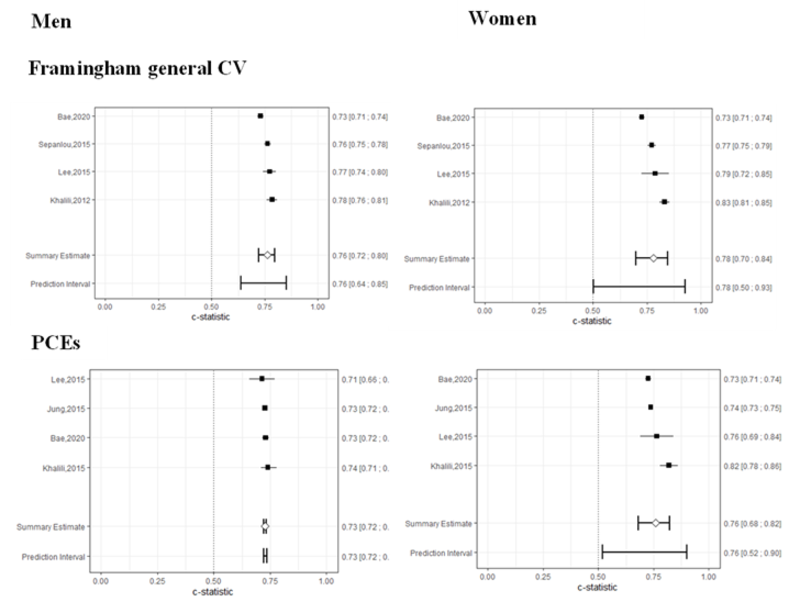

Supplement: S1 Fig — (TIF) [file pone.0292396.s002.tif]

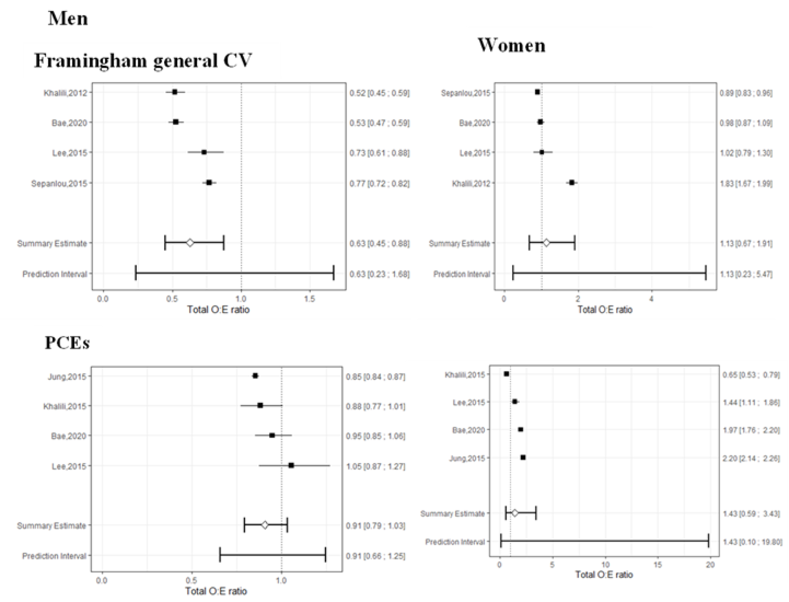

Supplement: S2 Fig — (TIF) [file pone.0292396.s003.tif]
