# Supplementary material for: Validity of the models predicting 10-year risk of cardiovascular diseases in Asia: A systematic review and prediction model meta-analysis
Source: PLoS One. 2023 Nov 30;18(11):e0292396. doi: 10.1371/journal.pone.0292396 (PMC10688732; doi:10.1371/journal.pone.0292396)
Supplement: S1 Table — (DOCX) [file pone.0292396.s004.docx]

**S1 Table.** Search strategy used to retrieve related documents

(Search date: November 28 2022)

| Search SYNTAX in PubMed | |
| --- | --- |
|  | ("Cardiovascular Diseases"[Mesh]) AND ("risk chart" OR "risk score" OR "risk equation" OR "risk algorithm" OR "risk prediction" OR "risk assessment") AND (validation OR calibration) AND (Asia OR "Middle east") |
| Search Syntax in Web of Science- conference proceedings citation index | |
|  | (ALL=("Cardiovascular Diseases") AND ALL=("risk chart" OR "risk score" OR "risk equation" OR "risk algorithm" OR "risk prediction" OR "risk assessment") AND ALL=(validation OR calibration) AND ALL=(Asia OR "Middle east")) |
| Search Syntax in Scopus | |
|  | (TITLE-ABS ("Cardiovascular Diseases"))  AND  ( TITLE-ABS ( "risk chart"  OR  "risk score"  OR  "risk equation"  OR  "risk algorithm"  OR  "risk prediction"  OR  "risk assessment" ) )  AND  ( TITLE-ABS (validation  OR  calibration ) )  AND  ( TITLE-ABS ( asia  OR  "Middle east" ) ) |
| Search Syntax in Open Access Theses and Dissertation (OATD) | |
|  | ("Cardiovascular Diseases") AND ("risk chart" OR "risk score" OR "risk equation" OR "risk algorithm" OR "risk prediction" OR "risk assessment") AND (validation OR calibration) AND (Asia OR "Middle east") |
| Search Syntax in Global Index Medicus | |
|  | tw:(("cardiovascular diseases") AND ("risk chart" OR "risk score" OR "risk equation" OR "risk algorithm" OR "risk prediction" OR "risk assessment") AND (validation OR calibration) ) |
