# Supplementary material for: Validity of the models predicting 10-year risk of cardiovascular diseases in Asia: A systematic review and prediction model meta-analysis
Source: PLoS One. 2023 Nov 30;18(11):e0292396. doi: 10.1371/journal.pone.0292396 (PMC10688732; doi:10.1371/journal.pone.0292396)
Supplement: S2 Table — (DOCX) [file pone.0292396.s005.docx]

**S2 Table** Summary of risk of bias analysis by PROBAST for included studies

| Author | Year | Risk model | Participants | Predictors | Outcome | Analysis | Overall |
| --- | --- | --- | --- | --- | --- | --- | --- |
| Liu | 2004 | Framingham CHD | Low | Low | Low | High | High |
| Jee | 2014 | Framingham CHD | Low | Low | Low | High | High |
| Bozorgmanesh | 2011 | Framingham general CVD | Low | Low | Low | High | High |
| Khalili | 2012 | Framingham general CVD | Low | Low | Low | High | High |
| Lee | 2015 | Framingham general CVD | Low | Unclear | Low | High | Unclear |
| Lee | 2015 | PCE | Low | Unclear | Low | High | Unclear |
| Jung | 2015 | PCE | Low | Low | Low | High | High |
| Khalili | 2015 | PCE | Low | Low | Low | High | High |
| Sepanlou | 2015 | Framingham General CVD | Low | Low | Low | High | High |
| Sawano | 2016 | SCORE | Low | Low | Low | High | High |
| Tang | 2019 | PCE | Low | Low | Low | High | High |
| Jiang | 2020 | Framingham General CVD | Low | Low | Low | High | High |
| Jiang | 2020 | PCE | Low | Low | Low | High | High |
| Fahimfar | 2022 | SCORE | Low | Low | Low | Low | Low |
| Fahimfar | 2022 | Globorisk | Low | Low | Low | Low | Low |
| Bae | 2020 | FRS | Low | Low | Low | High | High |
| Bae | 2020 | PCE | Low | Low | Low | High | High |
| Li | 2021 | WHO -laboratory-based | Low | Low | Low | High | High |
| Li | 2021 | WHO -Non-laboratoty-based | Low | Low | Low | High | High |
| Liu | 2022 | ACC/AHA 2013 PCE | Low | Low | Low | Low | Low |
| Tang | 2017 | ACC/AHA 2013 PCE | Low | Low | Low | High | High |
